# Infant-Directed Speech to 3-Month-Old Severe Preterm Infants: The Influence of Birth Weight and Maternal Depressive Symptoms

**DOI:** 10.3390/healthcare11121807

**Published:** 2023-06-20

**Authors:** Alessandra Provera, Erica Neri, Francesca Agostini

**Affiliations:** Department of Psychology “Renzo Canestrari”, University of Bologna, 40137 Bologna, Italy; erica.neri4@unibo.it (E.N.); f.agostini@unibo.it (F.A.)

**Keywords:** infant-directed speech, very low birth weight, extremely low birth weight, maternal postnatal depression

## Abstract

Severe premature birth (<32 weeks) is a risk factor for the development of maternal perinatal depression, while also affecting dyadic interactions and child outcomes. Although several studies have examined the impacts of prematurity and depression on early interactions, only a few studies have investigated the features of maternal verbal input. Furthermore, no study has investigated the relationship between the effect of severity of prematurity according to birth weight and maternal input. This study aimed to explore the effects of the severity of preterm birth and postnatal depression on maternal input during early interactions. The study included 64 mother–infant dyads, classified into three groups: 17 extremely low birth weight (ELBW) preterm infants, 17 very low birth weight (VLBW) preterm infants, and 30 full-term (FT) infants. At 3 months postpartum (corrected age for preterm infants), the dyads participated in a 5-min free interaction session. Maternal input was analyzed using the CHILDES system in terms of lexical and syntactic complexity (i.e., word types, word tokens, mean length of the utterance) and functional features. Maternal postnatal depression (MPD) was assessed using the Edinburgh Postnatal Depression Scale. The results showed that in high-risk conditions (i.e., ELBW preterm birth and maternal postnatal depression), maternal input was characterized by a lower frequency of affect-salient speech and a higher proportion of information-salient speech, specifically as directives and questions, suggesting that mothers in these conditions may experience more difficulty in conveying affective content to their infants. Moreover, the more frequent use of questions may reflect an interactive style characterized by a higher level of intrusiveness. These findings provide preliminary evidence of the impacts of prematurity severity and maternal depression on maternal verbal input, highlighting the importance of assessing both factors in clinical practice. Understanding the mechanisms underlying the impacts of prematurity and depression on early interactions may inform the development of tailored interventions aimed at promoting positive parent–infant interactions and child development.

## 1. Introduction

Prematurity is a condition that occurs in cases of infants born alive before the 37th week of pregnancy [1], and it could represent an important risk factor for the infant’s survival and development [2,3]. The risk of mortality or developmental delays is particularly evident when preterm infants are very small in terms of birth weight, as in cases of very low birth weight (VLBW; <1500 g) or extremely low birth weight (ELBW; <1000 g) conditions [2,4,5,6,7]. Several studies that focused on the neuropsychological trajectories of preterm infants have reported the presence of deficits or delays that could affect different developmental domains [8], such as the motor [9], cognitive [10], and linguistic domains [11,12,13]. Constituting a specific risk factor for the infant’s survival, health, and development, severe prematurity can represent a stressful and traumatic event for the parents, impairing the transition to parenthood, and leading to the exacerbation of negative feelings and perceptions about themselves as parents, along with recurrent and persistent worries about the baby [14]. Indeed, the literature underlines an increased risk of developing postnatal depressive and anxious symptomatology [15,16,17] in both mothers and fathers of high-risk preterm infants in the first postpartum period [18,19,20,21]. Such symptomatology can negatively affect both parenting and infant development [15,18], as it can impact on the mother’s ability to engage in interactive exchanges with her infant and reduce the quality and quantity of input directed towards the infant, potentially leading to more negative developmental outcomes [22,23].

Studies that analyzed the effect of the severity of prematurity on early dyadic interactions have highlighted a significant relationship between lower birth weight and the quality of mother–infant interactions in the first postpartum period [18,24]. Specifically, a study by Neri et al. [18] showed that ELBW preterm infants are more likely to have interactions characterized by high maternal intrusiveness and low remoteness. On the other hand, VLBW infants tend to have interactions with high levels of maternal sensitivity and infant communication. Furthermore, postnatal depression and anxiety in mothers have been associated with negative impacts on the quality of interactions, with depression being related to maternal remoteness and a negative affective state, and anxiety being linked to low sensitivity [18].

Infant-directed speech (IDS) represents a particular spontaneous register used by parents and caregivers to interact with infants and young children [25]. This form of speech is different from adult-directed speech (ADS), as it is characterized by linguistic and acoustic features such as grammatical simplification, lexical repetition, and exaggerated prosody [26]. These features have different important functions, such as arousing and sustaining the infant’s attention, facilitating dyadic interactions, conveying affective and informative contents, and promoting linguistic development [27,28,29,30]. Furthermore, IDS characteristics adjust dynamically over time, becoming more complex in order to support and shape the infant’s linguistic, cognitive, and socio-affective development [25,26,31]. Compared to ADS, speech’s verbosity is reduced in IDS [31]. Additionally, the amount of maternal speech generally decreases when addressed to younger infants and tends to increase as they get older, adjusting to the infants’ emerging skills [32,33].

As regards the lexical features, IDS is described as simpler, more concrete, and characterized by a lower variability in its semantic contents compared to ADS [31,34]. Vocabulary is more limited and characterized by a higher number of repetitions. Moreover, lexical features such as onomatopoeias, nonsense sounds, and a large use of diminutives are recurrent and typical of IDS, with an important affect-salient function, conveying affective contents during caregiver–infant interactions [27,31,33]. Simplifications in IDS also include its syntactic structures, which appear to be shorter and less complex compared to ADS [25,35]. The mean length of the utterance (MLU), which is a measure of syntactic complexity, is generally about three words in IDS and eight in ADS. Moreover, simple syntactic forms such as one-word utterances are frequent in IDS, but not in ADS [36,37].

Social–pragmatic aspects of IDS cover an important function in facilitating early dyadic interactions, because they allow infants and young children to infer the caregiver’s communicative or affective intent [25]. Interestingly, the literature highlights a variation in the functional characteristics of IDS across the first two years postpartum [24]. In the early stages of development, adult speech addressed to young infants is described as more affective and characterized by a higher proportion of affect-salient utterances, such as diminutives, proper names, and onomatopoeias [27,31,33]. As the infant grows up, IDS changes in its pragmatic functions, becoming more informative, contextualized, and descriptive [33,37]. The dynamic changes in the functional contents of IDS during the first year postpartum reflect the caregivers’ ability to adapt and adjust their speech according to the infants’ age and level of development [38].

As mentioned above, IDS plays a key role in promoting closeness and early social interactions between the caregiver and the infant during the first years. At the same time, both formal and functional aspects of maternal speech addressed to the infant are influenced by the quality of mothers’ affective states. Studies focused on depressed mothers have reported different IDS patterns and features compared to non-depressed mothers’ speech (for a review, see [39]). As regards the relations between IDS verbosity and maternal postnatal depression, several studies have reported a negative association between the amount of maternal IDS (in terms of total utterances, word tokens, and number of vocalizations) and the presence of maternal depressive symptomatology, highlighting the tendency of mothers with higher levels of depression to talk less with their babies. Conversely, a few studies [40,41] did not find any statistically significant differences in maternal speech’s verbosity between depressed and non-depressed mothers, suggesting the need for further studies. Concerning the lexical and syntactic complexity of maternal speech, measured by analyzing the number of different word types and the mean length of the utterance, Scheiber et al. [39] did not find in their review any statistically significant differences in IDS produced by depressed mothers compared to non-depressed ones. However, the authors warned about these results because of possible bias related to the different procedures and methods with which both depressive symptomatology and IDS were measured. An interesting finding emerged in a study by Reissland et al. [42], showing that depressed mothers, unlike non-depressed ones, did not show adaptation of their IDS with relation to the infant’s age. In fact, when talking to their infant, they did not use shorter utterances when the infant was younger (i.e., 6 months old) compared to at 10 months of age, while non-depressed mothers showed this change. The use of shorter and syntactically simpler utterances is prototypical of IDS directed towards younger infants, because their cognitive and pre-linguistic abilities are still limited. However, the lack of this aspect in depressed mothers could suggest a lower ability of mothers with depression to adapt the complexity of their IDS according to their infants’ age and abilities. 

Focusing on the contents of IDS, some studies have highlighted a significant relationship between the presence of depressed mood and the functional aspects of mothers’ speech addressed to the infant. Specifically, in their study, Herrera et al. [43] reported a negative association between the amounts of affect-salient speech and information-salient speech directed to 6-month-old infants and the presence of higher levels of maternal depressive symptomatology. 

As previously mentioned, previous studies have examined the characteristics of early mother–child interactions in cases of prematurity [18,24]. Of those, only a few studies examined the characteristics of communicative exchanges and infant-directed speech directed towards the population of preterm infants. For example, a study by Salerni et al. [44], which analyzed the characteristics of maternal IDS directed towards 6-month-old premature infants, did not detect significant differences compared to the syntactic and lexical complexity of input directed towards full-term infants. However, this study did not consider the severity of prematurity as a possible influencing factor. Furthermore, to date, there are no studies in the literature that have examined the features of communicative exchanges in the very first months postpartum.

As research has shown that both prematurity and maternal postnatal depression can have significant impacts on infant development, we could hypothesize that one of the factors involved in this effect might be the quality of maternal input addressed to the infant during the first years postpartum. 

In this paper, we explore the effects of the severity of prematurity (based on birth weight) and maternal postnatal depression on the lexical, syntactic, and functional features of infant-directed speech at 3 months postpartum. It is possible to hypothesize that the presence of higher-risk situations, such as severe prematurity and higher levels of maternal postnatal depression, may influence the input directed towards the infant. Specifically, these conditions might lead to the development of different interactive modalities, which could be expressed in terms of significant differences in the syntactic, lexical, or functional features of the maternal IDS.

## 2. Materials and Methods

### 2.1. Study Design

The present pilot, prospective, exposed and non-exposed study is part of a broader research and follow-up project that aims to investigate the effects of severe prematurity on infant developmental trajectories, the presence of parental postpartum symptomatology, and the quality of early dyadic exchanges during the first year postpartum.

### 2.2. Participants

A total of 64 mother–infant dyads participated in the study. Of these, 30 mothers of full-term (FT) infants (gestational age > 36 weeks, and birth weight > 2500 g) were recruited at the antenatal classes held in Cesena during the third trimester of pregnancy, while 34 were mothers of severely preterm infants (gestational age < 32 weeks, and birth weight < 1500 g), who had been hospitalized at the Neonatal Intensive Care Unit (NICU) of the Bufalini Hospital of Cesena (Italy). Preterm dyads were differentiated into two subgroups on the basis of the infants’ birth weight: 17 infants with a birth weight between 1500 and 1000 g were included in the very low birth weight (VLBW) group, and 17 infants with a birth weight less than 1000 g were included in the extremely low birth weight (ELBW) group. 

The exclusion criteria were the same for all of the groups, including the presence of infant neurological disease or complications, genetic syndromes or diseases, the presence of pre-existing maternal psychiatric disorders, multiple pregnancies, and lack of fluency in the Italian language.

Before starting the study, the research project obtained approval from the Ethics Committee of the University of Bologna (Protocol Number: 0001092/2023).

### 2.3. Procedure

Data were collected at 3 months postpartum (corrected ages were considered for preterm infants) at the Laboratory of Developmental Psychodynamics “Anna Martini” (Department of Psychology, University of Bologna). 

During the assessment, all participants were asked to provide a written consent form and to complete a sociodemographic questionnaire and the Edinburgh Postnatal Depression Scale (EPDS; [45]), so as to assess their postpartum mood; furthermore, a psychologist assessed the infants’ levels of mental and psychomotor development. Subsequently, all dyads were asked to participate in a free interaction session lasting about 5 min. All of the dyads were assessed in the same setting, which included the presence of puppets and toys suitable for the infants’ age. Each session was video recorded and, subsequently, all of the maternal utterances addressed to the infants were fully transcribed using the Codes for the Human Analysis of Transcripts (CHAT; [46]) format. 

### 2.4. Measures

#### 2.4.1. Sociodemographic Data

All mothers completed an ad hoc sociodemographic questionnaire, which included information about their age, years of education, type of delivery, marital status, and employment status. Perinatal data were also collected for all dyads. 

#### 2.4.2. Maternal Postnatal Depression (MPD)

The presence of maternal depressive symptomatology in the postpartum period was assessed by using the Edinburgh Postnatal Depression Scale (EPDS; [45]) self-report questionnaire. This questionnaire is one of the most widely used tools in international research for assessing maternal and paternal depression in the perinatal period. The instrument is composed of 10 items, scoring from 0 to 3, which evaluate the maternal mood over the previous 7 days. Higher total EPDS scores highlight the probable presence of increased levels of depression. In this study, the EPDS questionnaire was used as a categorical variable; as suggested by the validation study for the Italian version of the instrument [47], the cutoff value was set at 10 points in order to discriminate depressed mothers from non-depressed ones. 

#### 2.4.3. Infant Mental and Psychomotor Development

The Griffiths Mental Development Scales—Revised Version (GMDS-R for 0–2 years; [48]) were individually administered to evaluate the infants’ levels of mental and psychomotor development. These scales are a set of developmental assessments used to evaluate the cognitive and functional abilities of infants and young children in five specific areas (locomotor, personal and social, hearing and language, eye–hand coordination, and performance). The Griffiths scales are frequently used in clinical and research settings to aid in the diagnosis of developmental delays and to monitor progress over time in high-risk contexts such as preterm infants [7,10,18,19].

#### 2.4.4. Infant-Directed Speech

The total amount of maternal speech addressed to the infant was analyzed considering the number of utterances produced during the free interaction session. The utterance, which is any sequence of speech separated from the following utterance by a pause longer than 1 s [49], was considered as the unit of analysis.

The lexical and syntactic features of maternal input were examined by analyzing the following measures:

- Word tokens, i.e., the total number of words produced;
- Word types, i.e., the total number of different words produced;
- Mean length of utterance (MLU), i.e., the ratio of words to utterance, representing a measure of syntactic complexity.

The analysis of all of these variables was conducted using the CHILDES system [46], a specialized tool designed for the analysis of maternal and infant speech.

Functional characteristics of the maternal input were evaluated by using an ad hoc coding scheme previously used in studies on maternal speech (e.g., [50,51]). According to the scheme, each maternal utterance was attributed to one of the following mutually exclusive functional categories:

- Affect-salient speech, which includes utterances whose aim is to maintain the conversation (i.e., greeting, encouragement, singing);
- Information-salient speech, which includes utterances whose aim is to convey contents, giving or asking information. This functional category was included in the model, divided into four mutually exclusive subcategories: questions, labelling, descriptions, and directives;
- Attention getter, which includes utterances whose aim is to attract the infant’s attention (i.e., the infant’s name);
- Other, which includes incomplete or unintelligible utterances, or maternal speech that is not directed to the infant.

For each category and subcategory, the proportion of maternal speech in the total of maternal utterances produced during the interaction was computed.

### 2.5. Data Analysis

According to the objectives of the study, the main analysis strategy was to compare the characteristics of IDS according to the three birthweight categories, the absence/presence of depressive symptomatology, and the interaction between these two variables.

Preliminarily, the presence of differences between the three groups regarding both maternal and infant demographic characteristics was investigated by running a series of analyses of variance (ANOVAs). Specifically, the groups were compared in terms of maternal age, years of education, civil status, type of delivery, employment status, infant’s gender, infant’s (corrected) age, infant’s gestational age, and developmental quotient. The results revealed statistically significant differences between groups in terms of the mothers’ years of education, the type of delivery, and the infant’s gestational age. 

To assess the effects of birth status and postpartum depression on the characteristics of maternal input, a multivariate analysis of covariance (MANCOVA) was run. Both birth weight (FT, VLBW, and ELBW) and maternal depression (depressed and non-depressed) were set as between-subject factors, and speech characteristics (i.e., word types and tokens, MLU, proportion of affect-salient speech, directives, questions, labelling, descriptions, attention getter, and other) were set as dependent variables. 

A *p*-value < 0.05 was considered statistically significant.

Data analysis was conducted using IBM SPSS Statistics 24 software (IBM Corporation New York, NY, USA). 

## 3. Results

Maternal and infant descriptive characteristics are reported in Table 1. No statistically significant differences between groups emerged regarding maternal age, infant’s age, or infant developmental quotient. On the other hand, the level of education appeared to be significantly higher in FT infants’ mothers compared to ELBW ones (F (2, 61) = 4.78, *p* = 0.01; Bonferroni *post hoc* FT vs. ELBW, *p* = 0.02). The groups showed statistically significant differences regarding the infant’s gestational age at birth and the type of delivery. Specifically, in both the ELBW and VLBW groups compared to FT, cesarean delivery was more frequent than spontaneous delivery (X^2^ = 37.8, *p* = 0.00; Bonferroni *post hoc* ELBW vs. FT, *p* = 0.00; VLBW vs. FT, *p* = 0.00). Furthermore, gestational age was significantly smaller in the ELBW group compared to the VLBW and FT groups, and in the VLBW group compared to the FT group (F (2, 61) = 326; Bonferroni *post hoc* comparisons between all groups, *p* = 0.00). Since these two variables are strictly connected to prematurity, these results were expected. 

Analyzing the impacts of these three variables on IDS features, a significant effect emerged only for maternal years of education (F (9, 52) = 2.99, *p =* 0.01); therefore, this variable was included as a confounding variable in the subsequent analyses.

Sociodemographic differences between depressed and non-depressed mothers were also explored; no statistically significant differences emerged between the two groups.

As the mean years of education differed among the three groups, this variable was set as a covariate in the analysis of the variance of the features of maternal speech. Overall, multivariate analysis of covariance showed a statistically significant effect of maternal depression (Pillai’s trace: F (10, 48) = 2.15, *p* = 0.03, partial η^2^ = 0.28), but neither birth status (F (20, 98) = 1.02, *p* = 0.45, partial η^2^ = 0.12) nor the interaction between maternal depression and birth status (F (20, 98) = 0.66, *p* = 0.87, partial η^2^ = 0.11) was statistically significant. 

In terms of univariate analyses, no significant effects of either birth status or maternal depression emerged with respect to both the syntactic and lexical complexity of maternal speech (all *p*s > 0.05; Table 2). 

As regards the functional aspects of infant-directed speech (Table 3), data analysis highlighted the presence of statistically significant effects of both birth status and maternal depression, but not their interaction, on the proportion of affect-salient speech and questions addressed to the infant. Specifically, mothers of more severe preterm infants (i.e., ELBW) seemed to use less affect-salient speech (F (2, 49) = 3.60, *p* = 0.03) and more questions (F (2, 49) = 3.32, *p* = 0.04) when talking to their babies, compared to the mothers of VLBW and FT infants. It is interesting to note that a similar and statistically significant pattern was observed between depressed mothers and non-depressed ones (affect salient-speech: F (1, 49) = 10.83, *p* = 0.00; questions: F (1, 49) = 6.61, *p* = 0.01). Furthermore, the results showed a significant effect of maternal depression on the proportion of directives (F (1, 49) = 4.39, *p* = 0.04) addressed to the infant, as depressed mothers were more directive with their babies compared to non-depressed ones (Table 3).

No significant differences among groups were found regarding the other dependent variables.

## 4. Discussion

The aim of the present exploratory study was to investigate the potential impacts of the severity of preterm birth and maternal postpartum depression on the features of infant-directed speech addressed to FT and PT infants.

Despite previous research having underlined how both maternal postnatal depression and the severity of prematurity could impact on the quality of early mother–infant interactions [18,24], to the best of our knowledge, this is the first study to investigate their possible influence on IDS.

In general, the multivariate models showed significant results only regarding the effects of maternal depression on IDS characteristics. Given the exploratory nature of the research, and considering these as preliminary findings, we will discuss the significant results of the multivariate and univariate analyses as well, as they may serve as a promising starting point for future investigations.

Overall, our results showed that neither lexical nor syntactic aspects of IDS—in terms of word types, word tokens, and MLU—nor maternal speech verbosity were affected by birth status and/or maternal postnatal depression. In line with our results, previous studies on IDS directed to PT infants compared to FT ones also found no intergroup differences in the morphosyntactic and lexical features of maternal speech [44,52]. However, these studies did not take into account the severity of prematurity, which could be an important influencing factor, as observed in studies on interactive patterns [18,24]. The present study extends these findings by showing similarities in the structural complexity of maternal inputs directed to both VLBW and ELBW infants, as well as to typically developing infants at an earlier stage of development. However, as maternal speech modifies in accordance with the infant’s emerging skills, differences in IDS features may emerge later, in accordance with the infant’s communicative and linguistic abilities [51]. 

As emerged from the data analysis, the amounts of IDS were similar in depressed and non-depressed mothers. A recent review by Scheiber et al. [39] on the features of child-directed speech in cases of maternal postnatal depression reported that, overall, depressed mothers tend to speak less with their babies. However, two of the six studies included in the review, which analyzed the effects of maternal depression on speech verbosity, reported no significant differences in the mean number of utterances produced by depressed and non-depressed mothers [41]. The presence of conflicting results may be attributed to the multiple differences between the studies, regarding both the children’s age (the range varies from 3 to 42 months), and the measures and methodologies used to assess the quantity of maternal input and maternal depressive symptoms. Therefore, the existence of inconsistent findings regarding the impact of maternal depression on speech verbosity highlights the need for further research.

No significant effects of maternal depression emerged on the lexical and morphosyntactic features of IDS. Specifically, the syntactic and lexical complexity of IDS, as measured in terms of MLU and word types, appeared to be similar in depressed and non-depressed mothers. This result is consistent with the findings of previous studies; specifically, it is in line with the study by Murray et al. [53], conducted on infants of similar age and on mothers whose depressive symptomatology was detected by using the EPDS—the same tool used in our study. Moreover, studies on older infants showed similar results, suggesting that maternal perinatal depressive symptoms did not affect the lexical [54] or syntactic [55] complexity of maternal speech directed to the infant. 

Interestingly, when we explored the functional characteristics of IDS, our analyses showed a significant effect of birth status on the proportion of affect-salient speech and questions directed towards the infant. The results revealed that affect-salient speech constituted approximately half of the total utterances produced in both the FT and VLBW groups, but not in the ELBW group. Conversely, the proportion of interrogative sentences was significantly higher in the ELBW group compared to the others.

As the literature suggests, a higher proportion of affect-salient speech is expected in maternal inputs addressed to 3-month-old infants [27,33]. As highlighted by several perspectives (i.e., developmental psychology and psychopathology, infant research, affective neurosciences), during this developmental stage, maternal affective speech plays a crucial role in engaging the infant in positive interactions and promoting the development of a secure attachment bonding [24,56,57]. From the first weeks after childbirth, the baby is able to process emotional information during dyadic interactions and prone to recognizing maternal affective expressions, which are frequently conveyed through maternal input [58,59]. Research has indeed shown that younger infants are more responsive to affect-salient speech compared to information-salient speech, which becomes more relevant in later developmental stages, shaping the infant’s cognitive, linguistic, and communicative development [28]. Therefore, the maternal ability to convey emotional contents is crucial for enhancing the infant’s emotional and social development, in addition to co-regulating mother–infant interactions.

Conversely, maternal speech directed to ELBW infants seemed to be more demanding and characterized by a higher proportion of questions and a lower proportion of affect-salient speech. Premature infants have been frequently described as more passive and less involved in early dyadic interactions, as compared to typically developing infants [60,61]. Due to the increased passiveness of their children, ELBW infants’ mothers may use more interrogative sentences to elicit their engagement during interactive exchanges. The overstimulating communicative style adopted by ELBW mothers while speaking to their babies could also reflect a certain degree of intrusiveness in interaction. This hypothesis is coherent with a previous study by Agostini et al. [24], which reported more controlling and intrusive interactive behaviors in ELBW mothers compared to VLBW and FT ones. In fact, this interactive pattern would become evident when considering more severely premature infants, as a more intense maternal stimulation would emerge to enhance infant responsiveness [61]. However, it is uncertain whether these outcomes remain consistent over time; thus, future studies are needed to investigate whether this trend persists in the later stages of child development.

Regarding the influence of maternal depression on the functional characteristics of IDS, data analysis revealed a significant effect with regard to the proportion of affect-salient speech, and to the number of interrogative and directive sentences. Specifically, the input of depressed mothers was found to be characterized by lower affective salience and a higher number of interrogatives and directives. This result is consistent with a previous study by Herrera et al. [43] and may indicate a decreased ability of mothers with depressive symptoms to interact in a sensitive manner with their children, which means using more information-salient contents rather than affect-salient ones. Additionally, as higher information-salient input is more functional for sustaining the emerging linguistic and communicative skills in older children, the presence of more demanding maternal speech in depressed mothers may express a decreased ability to adapt their input to the their infant’s developmental level, which would, in turn, suggest a lower degree of sensitivity.

It is noteworthy that this study did not observe a significant interaction effect between severe prematurity and maternal depression, despite both being important factors that could influence maternal verbal input. This suggests that these factors may have independent impacts on the quality of maternal verbal input when considered individually. However, it is possible that other factors, which were not measured or controlled in this study (e.g., parenting stress, anxiety symptomatology, post-traumatic stress disorder symptoms), could affect the interaction between severe prematurity and maternal depressive symptoms. These findings highlight the importance of analyzing each factor separately when studying maternal verbal input in high-risk contexts, and of assessing multiple and different risk factors.

### Limitations and Future Directions

As mentioned above, this was an exploratory study aiming to investigate the influence of both maternal postnatal depression and the severity of prematurity on the features of IDS. One of the main limitations of this study is the limited sample size, which restricts the generalizability of its results and reduces the power of data analyses; therefore, future research would benefit from an increase in the number of subjects. Another limitation of this study is represented by the exclusion of fathers from the research design. The follow-up program offered by the Laboratory of Developmental Psychodynamics (Department of Psychology, University of Bologna) involves the participation of both parents but, due to the low number of fathers who agreed to participate, they were not included in the present study. Future research should consider the influence of both maternal and paternal affective states, as these might represent important risk or protective factors for child development and wellbeing, as suggested by the literature [23]. Understanding the impact of premature birth on both parents’ interactive behaviors can inform the development of targeted interventions and support programs for families in similar situations. A further limitation of our study is the collection of a limited range of sociodemographic variables; moreover, we could not collect data about the family history and obstetrics (such as corticotherapy, arterial umbilical pH, infant complications or diseases) of the participants in the study. For this reason, further studies should deepen our results by also including these variables and exploring any possible significant risk or protective effect in relation to the development of postpartum symptoms. An additional limitation of our study is represented by the use of a self-report questionnaire (EPDS) to assess the presence of maternal depressive symptoms. Future studies may benefit from complementing the use of self-report measures with diagnostic tools such as structured clinical interviews. Finally, since the literature has identified parents of severely premature children as being at higher risk of developing various forms of postpartum symptomology (e.g., anxiety, parenting stress, post-traumatic stress disorder symptoms), further studies should investigate the effects of other symptoms on IDS patterns.

## 5. Conclusions

Taken together, these results suggest that different adverse conditions during the perinatal period, such as the ones represented by maternal postnatal depression and severe premature birth, can have significant impacts on a mother’s communication style. This, in turn, can affect the mother’s ability to engage in developmentally appropriate and sensitive interactions with her infant. From a clinical perspective, this research highlights the relevance of providing psychological support during the perinatal period to mothers who are experiencing depressive symptomatology or who have given birth to a severely premature baby. This study also suggests how important it is to promote awareness and provide sufficient training to the hospital staff, in order to create an environment that supports early mother–infant interactions, especially acknowledging the relevant role played by maternal vocal input during the first few weeks postpartum. In this way, it becomes possible to identify potential risk factors for the baby’s and mother’s mental health, and to implement early interventions to support the parenting functions and foster mother–infant bonding.

## Figures and Tables

**Table 1:** Participants’ descriptive characteristics and differences between groups.

		FT (N = 30)	VLBW (N = 17)	ELBW (N = 17)	F/X^2^	*p*
**Maternal variables**	Chronological age	32.7 (5.63)	33.4 (5.52)	33.4 (5.63)	0.14	0.87
	Years of education	14.6 (2.94)	12.5 (3.0)	12.0 (3.45)	4.78	0.01
	Marital status ^a^				0.49	0.78
	Married	19 (63)	10 (59)	9 (53)		
	Other	11 (37)	7 (41)	8 (47)		
	Employment status ^a^				0.80	0.67
	Employed	26 (87)	13 (76)	14 (82)		
	Unemployed	4 (13)	4 (24)	3 (18)		
	Type of delivery ^a^				37.8	0.00
	Spontaneous delivery	27 (90.0)	6 (35.3)	4 (23.5)		
	Cesarean delivery	3 (10.0)	11 (64.7)	13 (76.5)		
**Infant variables**	Infant’s gender ^a^				4.63	0.09
	Male	10 (33.3)	11 (64.7)	9 (52.9)		
	Female	20 (66.7)	6 (35.3)	8 (47.1)		
	Infant’s age (in months) ^b^	2.7 (0.55)	3.01 (0.54)	2.97 (0.57)	2.23	0.11
	Gestational age at birth (in weeks)	40.17 (2.19)	29.76 (1.21)	27.05 (1.77)	326	0.00
	GMDS-R total score ^c^	113 (8.63)	112 (7.07)	107 (10.9)	2.73	0.07

Data are expressed as means (with standard deviations in parentheses) for interval data. ^a^ Number (and % in parentheses) for categorical data. ^b^ Corrected age for preterm infants. ^c^ Calculated based on corrected age for preterm infants.

**Table 2:** Mean, standard deviation, and univariate analyses of syntactic and lexical features of IDS.

	Birth Weight			Maternal Postnatal Depression		Birth Weight		Maternal Depression		BW X MPD	
	FT (N = 30)	VLBW (N = 17)	ELBW (N = 17)	Depressed (N = 23)	Non-Depressed (N = 41)	F	*p*	F	*p*	F	*p*
**Word tokens**	294.15 (19.29)	281.16 (25.55)	329.02 (23.64)	289 (20.98)	313.88 (16)	1.37	0.26	0.64	0.43	1.21	0.31
**Word types**	102.44 (6.06)	97.75 (8.02)	111.99 (7.42)	102.55 (6.59)	105.56 (5.02)	1.57	0.22	0.39	0.53	2.62	0.08
**MLU**	3.08 (0.12)	2.95 (0.16)	3.22 (0.15)	3.05 (0.13)	3.11 (0.10)	0.79	0.46	0.04	0.85	1.45	0.24

Data are expressed as estimated marginal means (with standard errors in parentheses) for interval data.

**Table 3:** Mean, standard deviation, and univariate analyses of functional features of IDS.

	Birth Weight			Maternal Depression		Birth Weight		Maternal Postnatal Depression		BW X MPD	
	FT (N = 30)	VLBW (N = 17)	ELBW (N = 17)	Depressed (N = 23)	Non-Depressed (N = 41)	F	*p*	F	*p*	F	*p*
**Affect-salient speech**	0.39 (0.03)	0.44 (0.04)	0.30 (0.04)	0.32 (0.03)	0.44 (0.02)	3.60	0.03	10.8	0.00	0.03	0.97
**Labelling**	0.01 (0.01)	0.01 (0.01)	0.03 (0.01)	0.02 (0.00)	0.02 (0.00)	2.37	0.10	0.06	0.81	0.24	0.80
**Descriptions**	0.20 (0.02)	0.16 (0.03)	0.18 (0.01)	0.20 (0.02)	0.16 (0.02)	2.29	0.11	1.29	0.26	1.08	0.35
**Questions**	0.28 (0.02)	0.29 (0.03)	0.37 (0.03)	0.35 (0.02)	0.28 (0.02)	3.32	0.04	6.61	0.01	0.90	0.41
**Directives**	0.05 (0.01)	0.05 (0.02)	0.06 (0.02)	0.06 (0.01)	0.04 (0.01)	0.59	0.56	4.39	0.04	0.09	0.91
**Attention getter**	0.04 (0.01)	0.03 (0.01)	0.03 (0.01)	0.03 (0.04)	0.03 (0.00)	0.10	0.90	0.01	0.93	0.62	0.54
**Other**	0.00 (0.00)	0.01 (0.01)	0.01 (0.01)	0.02 (0.00)	0.03 (0.00)	1.44	0.25	1.16	0.29	0.28	0.75

Data are expressed as estimated marginal means of the proportion of utterances in each category of functional speech (with standard errors in parentheses).

## Data Availability

The data presented in this study are available upon request from the corresponding author. The data are not publicly available due to privacy and ethical reasons.
